# Investigation for the efficacy of COVID-19 vaccine in Japanese CKD patients treated with hemodialysis

**DOI:** 10.1186/s41100-022-00427-2

**Published:** 2022-08-19

**Authors:** Ayumi Yoshifuji, Masataro Toda, Munekazu Ryuzaki, Kan Kikuchi, Toru Kawai, Ken Sakai, Emi Oyama, Masayoshi Koinuma, Kazuhiko Katayama, Yuki Uehara, Norio Ohmagari, Yoshihiko Kanno, Hirofumi Kon, Toshio Shinoda, Yaoko Takano, Junko Tanaka, Kazuhiko Hora, Yasushi Nakazawa, Naoki Hasegawa, Norio Hanafusa, Fumihiko Hinoshita, Keita Morikane, Shu Wakino, Hidetomo Nakamoto, Yoshiaki Takemoto

**Affiliations:** 1 Infection Control Committee, The Japanese Society for Dialysis Therapy, Tokyo, Japan; 2 Division of Nephrology, Department of Internal Medicine, Tokyo Saiseikai Central Hospital, Tokyo, Japan; 3 Faculty of Pharmaceutical Sciences, Teikyo Heisei University, Tokyo, Japan; 4 Laboratory of Viral Infection Control, Ōmura Satoshi Memorial Institute, Graduate School of Infection Control Sciences, Kitasato University, Tokyo, Japan

**Keywords:** COVID-19, Hemodialysis, Vaccination, Adverse reactions

## Abstract

**Background:**

Dialysis patients are predisposed to severe disease and have a high mortality rate in coronavirus disease 2019 (COVID-19) due to their comorbidities and immunocompromised conditions. Therefore, dialysis patients should be prioritized for vaccination. This study aimed to examine how long the effects of the vaccine are maintained and what factors affect antibody titers.

**Methods:**

Hemodialysis patients (HD group) and age- and sex-matched non-dialysis individuals (Control group), receiving two doses of BNT162b2 vaccine, were recruited through the Japanese Society for Dialysis Therapy (JSDT) Web site in July 2021. Anti-SARS-CoV-2 immunoglobulin (IgG) (SARS-CoV-2 IgG titers) was measured before vaccination, 3 weeks after the first vaccination, 2 weeks after the second vaccination, and 3 months after the second vaccination, and was compared between Control group and HD group. Factors affecting SARS-CoV-2 IgG titers were also examined using multivariable regression analysis and stepwise regression analysis (least AIC). In addition, we compared adverse reactions in Control and HD groups and examined the relationship between adverse reactions and SARS-CoV-2 IgG titers.

**Results:**

Our study enrolled 123 participants in the Control group (62.6% men, median age 67.0 years) and 206 patients in the HD group (64.1% men, median age 66.4 years). HD group had significantly lower SARS-CoV-2 IgG titers at 3 weeks after the first vaccination (*p* < 0.0001), 2 weeks after second vaccination (*p* = 0.0002), and 3 months after the second vaccination (*p* = 0.045) than Control group. However, the reduction rate of SARS-CoV-2 IgG titers between 2 weeks and 3 months after the second vaccination was significantly smaller in HD group than in Control (*p* = 0.048). Stepwise regression analysis revealed that dialysis time was identified as the significant independent factors for SARS-CoV-2 IgG titers at 2 weeks after the second vaccination in HD group (*p* = 0.002) and longer dialysis time resulted in higher maximum antibody titers. The incidences of fever and nausea after the second vaccination were significantly higher in the HD group (*p* = 0.039 and *p* = 0.020). Antibody titers in those with fever were significantly higher than those without fever in both groups (HD: *p* = 0.0383, Control: *p* = 0.0096).

**Conclusion:**

HD patients had significantly lower antibody titers than age- and sex-matched non-dialysis individuals over 3 months after vaccination. Dialysis time was identified as a factor affecting SARS-CoV-2 IgG titers in HD group, with longer dialysis time resulting in higher maximum SARS-CoV-2 IgG titers.

## Background

Coronavirus disease 2019 (COVID-19), caused by severe acute respiratory syndrome coronavirus type 2 (SARS-CoV-2), has become widespread worldwide, and the number of infected individuals is still increasing [1]. As hemodialysis (HD) patients have more comorbidities, and disordered immune function than normal subjects, chronic kidney disease or HD have been reported as risk factors for the severity of COVID-19. In fact, HD patients have an approximately 10 times higher mortality rate of COVID-19 than the general population [2]. Data until July 2021, when vaccine was not widely distributed in Japan, revealed that 383 of 1,349 patients (only with known outcome) died, with a high mortality rate of 28.4% [3]. Vaccines are considered very important for preventing serious conditions, leading to rapid vaccine distribution worldwide. The vaccines that have been mainly used in Japan thus far are the Pfizer-BioNTech vaccine (BNT162b2) and Takeda/Moderna vaccine (mRNA-1273). Both BNT162b2 and mRNA-1273 are highly effective in preventing the onset of severe diseases. The efficacy in the prevention of symptomatic onset of disease was reported to be 95% for the former and 94.1% for the latter 1 week after the second vaccination, and the efficacy in the prevention of severe disease was reported to be 89% for the former and 100% for the latter [1, 4]. In addition, known adverse reactions to vaccines include pain at the vaccination site, itching, general malaise, headache, chills, myalgia, arthralgia, and fever, although these symptoms generally improve within 2 days [1, 4]. The rates of allergic symptoms were 1.95% for BNT162b2 and 2.20% for mRNA-1273, and the rate of anaphylaxis was 4.7 per million patients for BNT162b2 and 2.5 per million patients for mRNA-1273 [5]. The vaccine was widely reported to be safe and effective, and the vaccination rate increased to approximately 80% in Japan. Dialysis patients are given priority for vaccination in Japan. As a result, the mortality rate for COVID-19 in dialysis patients until March 24, 2022, since August 2021 has decreased to 132 deaths (7.3%) out of 1,804 (only with known outcome) [3]. However, since HD patients have inadequate antibody acquisition and maintenance after vaccination with other vaccines such as influenza and hepatitis B, there is a concern that they may not be able to acquire and maintain sufficient antibodies through COVID-19 vaccination [6].

We aimed to investigate whether these patients have acquired an effective humoral response in neutralizing SARS-CoV-2, by quantitative analysis of immunoglobulin G (IgG) antibody titers to SARS-CoV-2 spike protein, which shows a strong correlation with neutralization [7, 8]. Furthermore, this study aimed to examine how long the effects of the vaccine are maintained and what factors affect antibody titers.

## Materials and methods

We conducted a prospective multicenter study by Infection Control Committee of the Japanese Society for Dialysis Therapy (JSDT). After receiving approval from the Ethics Committee of the JSDT (approval numbers 1-10), facilities that could recruit patients to participate in the study were enrolled from July 6 to July 31 on the JSDT homepage.

The conditions for enrollment for HD patients (HD group) were the following: subjects who had not yet been vaccinated or had received only one dose of the vaccine, had not been infected with COVID-19, had not been treated for any malignancy within 1 year, and had not been treated with drugs such as steroids, immunosuppressants, and immunomodulators, were scheduled to receive two doses of BNT162b2 vaccine and had given written consent for this study. Control group was registered by open recruitment at Tokyo Saiseikai Central Hospital and its affiliated facilities by matching the number of enrolled dialysis patients in terms of age (in 10-year increments) and sex. As with the HD group, Control group also consisted of patients who fulfilled the conditions set for HD patients, in addition to having an eGFR of 45 mL/min/1.73 m^2^, or more.

SARS-CoV-2 IgG antibody titers to the S1 subunit of the spike protein of SARS-CoV-2 (anti-S1 antibody titers) were measured using the Ortho-Clinical Diagnostics VITROS® Anti-SARS-CoV-2 IgG Chemiluminescent Immunoassay correlated with neutralizing antibodies at the following points before the first vaccination, 3 weeks after the first vaccination, 2 weeks after the second vaccination, and 3 months after the second vaccination. Patients with symptomatic COVID-19 during the study period were excluded. Patients with a baseline value of 17.8 BAU/mL (criteria for positive antibody titer) or higher prior to the first vaccination and those whose antibody titer 3 months after the second vaccination was higher than that of 2 weeks after the second vaccination were also excluded. The antibody titers over time were compared between the two groups. The patients were also classified into two groups: those with an antibody titer of less than 17.8 (non-responders) and those with an antibody titer of 17.8 or higher (responders) 3 weeks after the first vaccination. The percentage and the backgrounds and characteristics of non-responder were evaluated. Patient clinical characteristics (age, sex, Body Mass Index (BMI), primary kidney disease, comorbidities, HD vintage, dialysis time, Kt/V) and clinical data (total protein (TP), albumin (Alb), aspartate aminotransferase (AST), alanine aminotransferase (ALT), alkaline phosphatase (ALP), γ-glutamyl transpeptidase (γ-GTP), blood urea nitrogen (BUN), creatinine (Cre), uric acid (UA), immunoglobulin (IgG), iron (Fe), total iron binding capacity (TIBC), ferritin, C-reactive protein (CRP), hemoglobin A1c (HbA1c), white blood cell (WBC), lymphocyte, and hemoglobin (Hb)) were also collected. Factors affecting antibody titers at 2 weeks after the second vaccination and reduction rate (reduction rate was calculated by the differences of anti-S1 antibody titers between 2 weeks and 3 months after the second vaccination divided by those at 2 weeks after the second vaccination) were also evaluated.

In addition, neutralizing antibodies were measured at 3 months after the second vaccine by iFlash-2019-nCoV NAb surrogate neutralization assay (Yhlo-NAb; Shenzhen YHLO Biotech Co., Ltd., Shenzhen, China) to confirm the strong positive correlation with anti-S1 antibody titers.

Furthermore, a questionnaire survey was conducted after the first and second vaccinations to determine the presence of adverse reactions (pain, redness, swelling, pruritus, fatigue, headache, muscle pain, coldness, fever (37.5 °C <), arthralgia, nausea, diarrhea, stomachache, and anaphylaxis), which is compared between Control group and HD group. Then, we investigated the relation of adverse reaction with anti-S1 antibody titers in each group.

We approximated anti-S1 antibody titers at 2 weeks after the second vaccination and the reduction rate from 2 weeks to 3 months after the second vaccination to a normal distribution by sinh-arcsinh (SHASH) transformation and performed univariate analysis using background, comorbidities, and laboratory data as explanatory variables. Then, multivariable regression analysis was performed by extracting only factors with *p* values less than 0.25 in that univariate analysis, and then stepwise regression analysis (least AIC), using the JMP software program.

The median values were compared using the Mann–Whitney *U* test. Frequencies between groups were compared using Fisher’s exact test or the chi-square test. Statistical significance was set at *p* < 0.05.

## Results

**Fig. 1:**
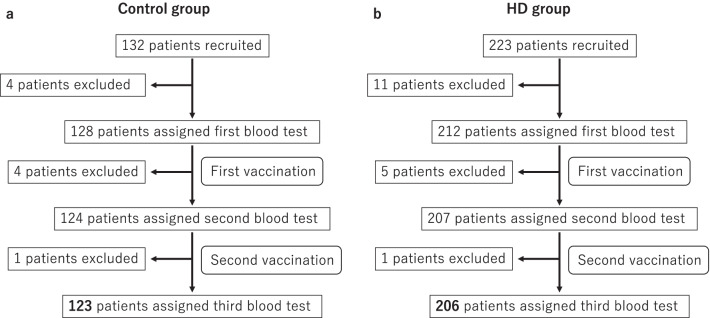
Trial profile. As control group, 132 patients were recruited and 4 patients were excluded before first blood test because we could not obtain consent (one patient), one had immunomodulate drugs (one patient), and they could not come to our hospital (two patients). Then, 4 patients were excluded before second blood test because one had COVID-19 (one patient), one could not come to our hospital (one patient), and they started immunomodulate drugs (two patients). One patient died before third blood test. Finally 123 patients were enrolled (**a**). As HD group, 223 patients were enrolled and 11 patients were excluded before first blood test because we could not obtain consent (five patients), they could not come to our hospital (two patients), they did not have vaccine or have other vaccine (three patients), and one died (one patient). Then, 5 patients were excluded before second blood test because they had antibody titer over 17.8 BAU/ml before first vaccination (three patients) or died (two patients). One patient died before third blood test. Finally 206 patients were enrolled (**b**). HD, hemodialysis

In this study, 10 facilities (Tokyo Saiseikai Central Hospital, Harada Naika Clinic, Ozawa Clinic, Mizuno Clinic, Nakamura Clinic, Konan-no-sato, Shirogane-no-mori, Keifukuen, Oumori Nursing Home, and Kurara-Kaminoge) participated as Control group and 7 facilities (Shinagawa Dialysis Clinic, Meguro Station Building Clinic, Tokyo Saiseikai Central Hospital, Omiya Yoshizawa Clinic, Urawa Yoshizawa Clinic, Minami-Ooi Clinic, and Chuou Naika Clinic) as HD group. In total, 132 participants were recruited as Control and 223 patients were recruited as HD group. In the Control group, 4 patients were excluded before first blood test because we could not obtain consent (one patient), one had immunomodulate drugs (one patient) and they could not come to our hospital (two patients). Then, 4 patients were excluded before second blood test because one had COVID-19 (one patient), one could not come to our hospital (one patient) and they started immunomodulate drugs (two patients). One patient was excluded before third blood test because the person died (Fig. 1a). In HD group, 11 patients were excluded before first blood test because we could not obtain consent (five patients), they could not come to our hospital (two patients), they did not have vaccine or have other vaccine (three patients), one died (one patient). Then, 5 patients were excluded before second blood test because they had antibody titer over 17.8 BAU/ml before first vaccination (three patients) or died (two patients). One patient was excluded before third blood test because the person died (Fig. 1b). Finally, 123 participants in Control group (62.6% men, median age 67.0 years) and 206 patients in HD group (64.1% men, median age 66.4 years) were enrolled (Fig. 1). The characteristics of each group are shown in Table 1.

**Table 1:** Characteristics of subjects

	Control group (*n* = 123)			HD group (*n* = 206)		
Sex (*n*, (%))	All	Male 77 (62.6)	Female 46 (37.4)	All	Male 132 (64.1)	Female 74 (35.9)
Age (years)	67.0	66.6	67.6	66.4	66.2	66.6
BMI	23.7	24.1	23.1	22.5	22.7	22.1
Creatinine (mg/dL)	0.84	0.92	0.71	10.99	11.51	10.08
Diabetes mellitus (*n*, (%))	17 (16.3)*	13 (20.0)	4 (10.3)	90 (43.7)	65 (49.3)	25 (33.8)
Hypertension (*n*, (%))	47 (45.2)*	33 (50.8)	14 (35.9)	84 (40.8)	56 (42.4)	28 (37.8)
Malignant tumor (*n*, (%))	10 (9.6)*	8 (12.3)	2 (5.1)	29 (14.1)	18 (13.6)	11 (14.9)
Cerebrovascular disease (*n*, (%))	6 (5.8)*	5 (7.7)	1 (2.6)	44 (21.4)	32 (24.2)	12 (16.2)
Cardiovascular disease (*n*, (%))	4 (3.8)*	3 (4.6)	1 (2.6)	41 (19.9)	29 (22.0)	12 (16.2)
COPD (*n*, (%))	9 (8.7)*	7 (10.8)	2 (5.1)	12 (5.8)	3 (2.3)	9 (12.2)

BMI, body mass index; COPD, chronic obstructive pulmonary disease; HD, hemodialysis

*n = 104 due to unavailability of data for 19 patients

### Anti-S1 antibody titers and contributing factors

**Fig. 2:**
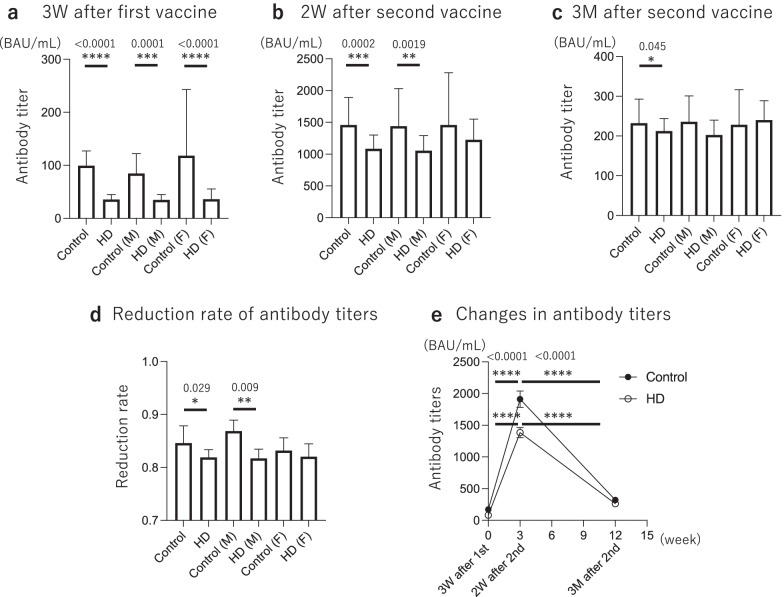
Antibody titers at each point after vaccination. HD group had significantly lower anti-S1 antibody titers before the second vaccination (HD: 35.6 BAU/mL vs Control: 99.4 BAU/mL, *p* < 0.0001), 2 weeks after the second vaccination (HD: 1,085 BAU/mL vs Control: 1,460 BAU/mL, *p* = 0.0002), and 3 months after the second vaccination (HD: 212.3 BAU/mL vs Control: 232.2 BAU/mL, *p* = 0.045) than the Control group (**a**–**c**). In addition, significant differences were observed 3 weeks after the first vaccination and 2 weeks after the second vaccination for males, and only 3 weeks after the first vaccination for females (**a**–**c**). Furthermore, from 2 weeks to 3 months after the second vaccination, the reduction rate of antibody titers (from 2 weeks to 3 months) was significantly smaller in HD group than in Control (HD: 0.819 vs Control: 0.846, *p* = 0.029), and, according to sex, only significant in male (**d**). **e** Time course of anti-S1 antibody titers over 3 months. *p < 0.05, **p < 0.01, ***p < 0.001, ****p < 0.0001. 3 W, 3 weeks; 2 W, 2 weeks; 3 M, 3 months; HD, hemodialysis; M, male; F, female; BAU, binding antibody unit

HD group had significantly lower anti-S1 antibody titers at 3 weeks after the first vaccination (HD: 35.6 BAU/mL vs Control: 99.4 BAU/mL, *p* < 0.0001), 2 weeks after second vaccination (HD: 1,085 BAU/mL vs Control: 1,460 BAU/mL, *p* = 0.0002), and 3 months after second vaccination (HD: 212.3 BAU/mL vs Control: 232.2 BAU/mL, *p* = 0.045) than Control group (Fig. 2a–c). When compared between the two groups after categorizing them by sex, in males, HD group had significantly lower anti-S1 antibody titers than Control group at 3 weeks after the first vaccination (HD: 34.8 BAU/mL vs Control: 84.6 BAU/mL, *p* = 0.0001) and 2 weeks after the second vaccination (HD: 1,055 BAU/mL vs Control: 1,440 BAU/mL, *p* = 0.0019), but there was no significant difference at 3 months after the second vaccination (HD: 202.7 BAU/mL vs Control: 235.9 BAU/mL, *p* > 0.05) (Fig. 2a–c). In females, the HD group had significantly lower anti-S1 antibody titers than the Control group at 3 weeks after the first vaccination (HD: 36.2 BAU/mL vs Control: 118.0 BAU/mL, *p* < 0.0001), but there were no significant differences at 2 weeks after the second vaccination (HD: 1,225 BAU/mL vs Control: 1,460 BAU/mL, *p* > 0.05), nor at 3 months after the second vaccination (HD: 240.1 BAU/mL vs Control: 228.1 BAU/mL, *p* > 0.05) (Fig. 2a–c). In addition, the reduction rate of anti-S1 antibody titers (from 2 weeks to 3 months) was significantly smaller in HD group than in Control (HD: 0.819 vs Control: 0.846, *p* = 0.029). Furthermore, when the reduction rate of anti-S1 antibody titers of the two groups was compared according to sex, it was smaller in the HD group, but only significant in males (Male HD: 0.817 vs Control: 0.869, *p* = 0.009; Female HD: 0.820 vs Control: 0.832, *p* > 0.05) (Fig. 2d). The time course of anti-S1 antibody titers from 3 weeks after the first vaccination to 3 months after the second vaccination is shown in Fig. 2e; the Control obtained higher antibody titers than the HD group after the second vaccination, but the antibody titer declined faster in the Control from 2 weeks to 3 months after the second vaccination, and the difference in antibody titer between the two groups became smaller.

HD patients had significantly higher proportion of non-responders compared to Control group at 3 weeks after the first vaccination (HD group 26.7% (55/206) vs Control group 13.8% (17/123), *p* = 0.0082). However, the majority of non-responders 3 weeks after the first vaccination in each group developed an immune response at 2 weeks after the second vaccination, and the proportion of non-responders significantly decreased (HD group 1.9% (4/206) vs Control group 1.6% (2/123), *p* = 1.0000). Among those who were non-responders after the first vaccination, antibody titers at 2 weeks after the second vaccination were significantly lower in the HD group than in the Control group (HD group 376.5 BAU/mL vs responders 538.0 BAU/mL, *p* = 0.038) (Table 2). Comparison of the non-responders and responders in HD patients showed no significant differences in age, sex, comorbidities, dialysis vintage, or Kt/V between the two groups, but antibody titers were significantly lower in non-responders at 2 weeks after the second vaccination (non-responders 376.5 BAU/mL vs responders 1,380 BAU/mL, *p* < 0.0001) and at 3 months after the second vaccination (non-responders 74.6 BAU/mL vs responders 257.7 BAU/mL, *p* < 0.0001) (Table 2).

**Table 2:** Percentage of responder after first vaccination and subsequent antibody titers

	Responder (%)		2 weeks after the second vaccination (BAU/mL)	3 months after the second vaccination (BAU/mL)
Control	86.2*	Non-responder (*n* = 17)	538.0	128.6
		Responder (*n* = 106)	1690.0	236.5
		*p *value	*p* < 0.0001	0.065
HD	73.3*	Non-responder (*n* = 55)	376.5	74.6
		Responder (*n* = 151)	1380	257.7
		*p* value	*p* < 0.0001	*p* < 0.0001

HD, hemodialysis

*p = 0.0082

### Neutralizing antibody titers

**Fig. 3:**
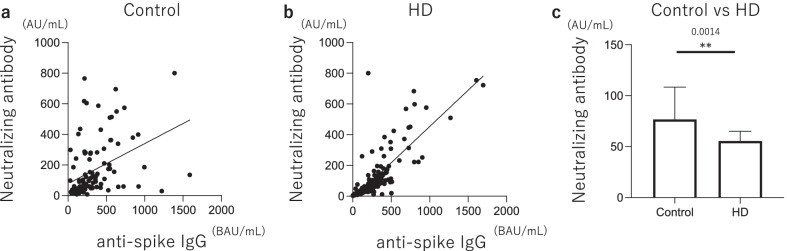
Neutralizing antibody 3 months after the second vaccination. The neutralizing antibody titers showed a strong positive correlation with anti-S1 antibody titer both in HD group and in Control group (HD: *r* = 0.81, *p* < 0.0001, Control: *r* = 0.40, *p* < 0.0001) (**a**, **b**). Also, HD group had lower neutralizing antibody titers at 3 months after the second vaccination (HD: 55.6 AU/mL vs Control: 76.7 AU/mL, *p* < 0.0014) (**c**). **p < 0.01. HD, hemodialysis; AU, antibody unit; BAU, binding antibody unit

The neutralizing antibody titers showed a strong positive correlation with the anti-S1 antibody titers both in Control and HD groups (HD: *r* = 0.81, *p* < 0.0001, Control: *r* = 0.40, *p* < 0.0001) (Fig. 3a, b). Also, the HD group had lower neutralizing antibody titers at 3 months after the second vaccine (HD: 55.6 AU/mL vs Control: 76.7 AU/mL, *p* < 0.0014) (Fig. 3c).

### Multivariable regression analysis of anti-S1 antibody titers

- Two weeks after the second vaccination

In the Control group, age, diabetes mellitus (DM), chronic obstructive pulmonary disease (COPD), BUN, and cerebrovascular disease were extracted in a univariate analysis. Although there were no significant independent factors for anti-S1 antibody titers in multivariable regression analysis, age and DM were identified significant independent factors for anti-S1 antibody titers in stepwise regression analysis (Table 3). In the HD group, dialysis time, age, BMI, hypertension, cardiovascular disease, COPD, BUN, and IgG were extracted in a univariate analysis, and patients with shorter dialysis time (less than 4 h) had significantly lower anti-S1 antibody titers in multivariable regression analysis and stepwise regression analysis (Table 4).

- The reduction rate from 2 weeks to 3 months after the second vaccination

**Table 3:** Multivariable analysis of factors affecting antibody titers in the Control group

Variable	Estimate	Standard variation	*p* value	VIF
*Antibody titer at 2 weeks (multivariable regression analysis)*				
Age	− 0.01	0.01	0.15	1.18
DM	− 0.20	0.12	0.10	1.05
COPD	0.32	0.19	0.09	1.02
BUN	− 0.02	0.02	0.27	1.20
Cerebrovascular disease	− 0.30	0.23	0.19	1.02
*Antibody titer at 2 weeks (stepwise regression analysis)*				
Age	− 0.02	0.01	**0.005**	1.00
DM	− 0.26	0.12	**0.04**	1.02
COPD	0.32	0.16	0.05	1.02
*Reduction rate of antibody titer (multivariable regression analysis)*				
BMI	0.06	0.03	0.05	1.12
Alb	− 0.60	0.38	0.12	1.61
Sex	− 0.06	0.14	0.69	1.22
Age	− 0.01	0.01	0.44	1.22
CRP	0.09	0.63	0.89	1.44
IgG	− 0.0001	0.0004	0.88	1.51
UA	0.10	0.10	0.29	1.15
*Reduction rate of antibody titer (stepwise regression analysis)*				
BMI	0.07	0.03	**0.03**	1.00
Alb	− 0.49	0.30	0.11	1.00

VIF, variance inflation factor; DM, diabetes mellitus; COPD, chronic obstructive pulmonary disease; BUN, blood urea nitrogen; BMI, body mass index; Alb, albumin; CRP, C-reactive protein; IgG, immunoglobulin G; UA, uric acid;

Statistically significant parts with *p* < 0.05 were shown in bold

**Table 4:** Multivariable analysis of factors affecting antibody titers in HD patients

Variable	Estimate	Standard variation	*p* value	VIF
*Antibody titer at 2 weeks (multivariable regression analysis)*				
Dialysis time	0.41	0.20	**0.04**	1.09
Age	− 0.01	0.01	0.26	1.10
BMI	0.02	0.02	0.22	1.08
Hypertension	0.10	0.07	0.14	1.05
Cardiovascular disease	− 0.09	0.09	0.29	1.11
COPD	0.21	0.14	0.14	1.06
BUN	− 0.004	0.004	0.41	1.08
IgG	0.0002	0.0002	0.31	1.12
*Antibody titer at 2 weeks (stepwise regression analysis)*				
Dialysis time	0.57	0.18	**0.002**	1.00
Hypertension	0.11	0.07	0.11	1.03
COPD	0.21	0.14	0.14	1.04
*Reduction rate of antibody titer (multivariable regression analysis)*				
COPD	0.24	0.13	0.06	1.03
Hypertension	0.12	0.06	0.05	1.05
Malignant tumor	0.12	0.09	0.18	1.02
BUN	− 0.01	0.004	0.06	1.03
CRP	− 0.12	0.10	0.22	1.01
*Reduction rate of antibody titer (stepwise regression analysis)*				
COPD	0.24	0.13	0.06	1.03
Hypertension	0.13	0.06	**0.04**	1.04
BUN	− 0.008	0.01	0.05	1.02

VIF, variance inflation factor; IgG, immunoglobulin G; COPD, chronic obstructive pulmonary disease; BUN, blood urea nitrogen; Alb, albumin; UA, uric acid

Statistically significant parts with *p* < 0.05 were shown in bold

In the Control group, BMI, albumin, sex, age, CRP, IgG, and UA were extracted in a univariate analysis. Although there were no significant independent factors for the decrease in anti-S1 antibody titers in multivariable regression analysis, BMI was identified significant independent factor for the reduction rate in stepwise regression analysis (Table 3). In the HD group, COPD, hypertension, malignant tumor, BUN, and CRP were extracted in a univariate analysis. Although there is no significant independent factor of reduction rate in multivariable regression analysis, hypertension was identified as a significant independent factor of the reduction rate in stepwise regression analysis (Table 4).

### Adverse reactions after vaccination

**Fig. 4:**
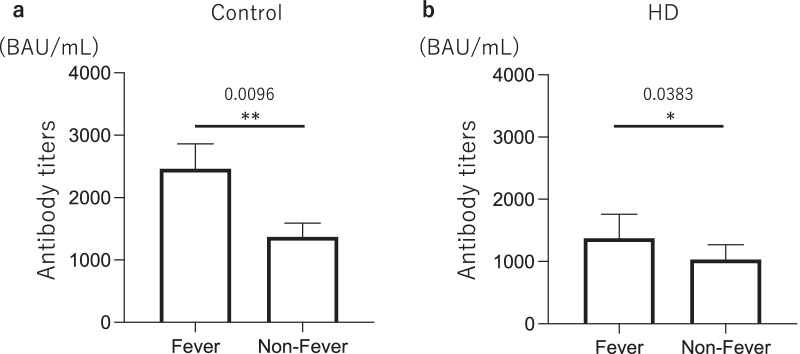
Antibody titers between patients with or without fever after the second vaccination. Anti-S1 antibody titers of subjects who developed fever 2 weeks after the second vaccination were significantly elevated than those who did not in both Control group and HD group (Control group: fever: 2,460 /mL vs non-fever: 1,370 BAU/mL, *p* = 0.0096, HD group: fever: 1,375 /mL vs non-fever: 1,030 BAU/mL, *p* = 0.0383). *p < 0.05, **p < 0.01. HD, hemodialysis; BAU, binding antibody unit

There was no significant difference in the incidence of adverse reactions after vaccination between two groups after the first vaccination; however, the incidences of fever and nausea were significantly higher in the HD group (*p* = 0.039 and *p* = 0.020, respectively) after the second vaccination. When the two groups were further compared according to sex, in males, the incidences of fever and nausea were significantly higher in the HD group (*p* = 0.026 and *p* = 0.046, respectively), only after the second vaccination. However, in women, there was no significant difference after the second vaccination (Table 5). Almost all patients who experienced nausea after the second vaccination also had fever; therefore, we compared anti-S1 antibody titers between the two groups of patients who had fever after the second vaccination and those who did not. As a result, anti-S1 antibody titers were significantly higher in the fever group 2 weeks after the second vaccination both in Control group (fever: 2,460 /mL vs non-fever: 1,370 BAU/mL *p* = 0.0096) and in HD group (fever: 1,375 /mL vs non-fever: 1,030 BAU/mL *p* = 0.0383) (Fig. 4).

**Table 5:** Adverse reactions after vaccination

		Local				Systemic									
		Pain	Redness	Swelling	Pruritus	Fatigue	Headache	Muscle pain	Coldness	Fever	Arthralgia	Nausea	Diarrhea	Stomachache	Anaphylaxis
*All*															
First vaccine	Control (*n* = 121)	67	11	14	15	23	7	32	2	7	3	0	3	2	0
	HD (*n* = 208)	107	13	19	19	24	5	52	3	5	7	5	5	1	0
	*p value*	0.49	0.34	0.48	0.35	0.06	0.11	0.77	0.88	0.11	0.65	0.09	0.97	0.28	> 0.99
Second vaccine	Control (*n* = 121)	61	12	17	24	27	14	30	4	21	5	0	0	0	0
	HD (*n* = 208)	109	15	21	26	50	23	52	16	57	16	9	4	4	0
	*p value*	0.73	0.39	0.28	0.07	0.72	0.89	0.97	0.11	**0.039**	0.20	**0.020**	0.12	0.12	> 0.99
*Male*															
First vaccine	Control (*n* = 74)	40	4	5	5	10	5	15	0	4	1	0	3	2	0
	HD (*n* = 134)	62	5	5	9	11	3	35	2	5	6	2	2	1	0
	*p value*	0.28	0.56	0.32	0.71	0.22	0.11	0.35	0.29	0.57	0.23	0.29	0.25	0.26	> 0.99
Second vaccine	Control (*n* = 74)	34	4	4	9	14	7	14	1	10	4	0	0	0	0
	HD (*n* = 134)	65	7	9	12	26	12	33	9	36	14	7	2	3	0
	*p value*	0.72	0.96	0.71	0.46	0.93	0.90	0.35	0.08	**0.026**	0.21	**0.046**	0.29	0.19	> 0.99
*Female*															
First vaccine	Control (*n* = 46)	27	7	9	10	13	2	17	2	3	2	0	0	0	0
	HD (*n* = 73)	45	8	14	10	13	2	17	1	0	1	3	3	0	0
	*p value*	0.75	0.50	0.96	0.25	0.18	0.63	0.11	0.31	**0.027**	0.31	0.16	0.16	> 0.99	> 0.99
Second vaccine	Control (*n* = 46)	27	8	13	15	13	7	16	3	11	1	0	0	0	0
	HD (*n* = 73)	44	8	12	14	24	11	19	7	21	2	2	2	1	0
	*p value*	0.86	0.32	0.12	0.10	0.60	0.98	0.31	0.56	0.56	0.85	0.26	0.26	0.43	> 0.99

HD, hemodialysis

Statistically significant parts with *p* < 0.05 were shown in bold

## Discussion

Various studies have been conducted on vaccine antibody titers in HD patients, but most studies have involved healthcare workers as a control group who were not age- and sex-matched. This study provides a significant contribution to this problem because antibody titers were compared between Control and HD patients in age- and sex-matched conditions.

Previous reports have shown that 93–95% of the general population had elicited a humoral immune response 3–4 weeks after the first immunization, but only 18–43% of HD patients had elicited a humoral immune response [9–11]. In our study, the proportion of HD patients who developed positive antibody titers after the first vaccination was 74%, which was significantly lower than that of non-HD patients (86%), but higher than that of the previous report. Control group may have been affected by the fact that this study included even elderly patients who were age- and sex-matched with the HD patients. The results of HD group may have been caused by the fact that the Japanese race and the efficiency of Japanese HD could affect antibody titers, compared to previous reports.

Both Control and HD groups elicited a humoral immune response 2 weeks after the second vaccination, when antibody titers were expected to increase at the highest level, although there was a significant difference between the two groups. These data are consistent with previous reports showing that antibody titers increased significantly in 82% to 96% of cases 1 month after the second immunization [10–12]. However, although previously reported data showed that the antibody titers in HD patients were 40% of those in non-HD patients, our age-matched data showed 66% of those in non-HD patients. This is suspected to be due to Control group being an age-matched population of HD patients, unlike previous reports. Kageyama et al. listed immunosuppressive drug use, age, time between second dose and sample collection, glucocorticoid use, and alcohol consumption as factors contributing to this maximum antibody titer in non-HD patients [13]. We identified age and DM as significant independent factors. As patients with malignancies or immunomodulating drugs were excluded in our study, some factors Kageyama et al. listed were not identified. DM was also identified as the important factor to affect the antibody level because of the immune defects caused by hyperglycemia and insulin resistance [14]. Factors which affect the antibody levels were somewhat different in each study; therefore, integrated data in the form of systematic review would be important for further analysis.

As factors contributing to this maximum antibody titer in HD patients, Agur et al. identified younger age, higher albumin levels, lower intravenous iron doses, and BMI less than 30 [14] and Lacson et al. also identified women, younger age, immunosuppressed status due to disease or medications, chronic heart failure, and history of other vaccinations or hospitalizations before and after vaccination [15]. In our study, dialysis time was identified as factors affecting antibody titers. Among the dialysis group, those with shorter dialysis time have difficulty obtaining antibody titers. As previous reports, both the innate and adaptive immune systems are disturbed by uremia, leading to decreased antigen processing and reduced cell-mediated and antibody-mediated immune responses in HD patients [16]. Therefore, eliminating uremic toxins by daily dialysis for longer periods of time may lead to higher antibody titers.

It has been reported that vaccine antibody titers decline over time in dialysis patients [6]. Davidovic et al. reported that antibody titers declined to baseline level at 6 months after the first vaccination, indicating the importance of booster vaccination [17]. In this study, the factors that determine a faster decline in antibody titers were also investigated as reduction rate. To the best of our knowledge, this is the first study to evaluate the affecting factors using multivariate analysis and stepwise regression analysis. We found the factor which is associated with a decline in anti-S1 antibody titers in the Control group; BMI. Pellini et al. demonstrated that BMI affected the antibody titers in response to COVID‐19 vaccine because of the immune dysfunction [18]. However, no reports have been provided on the association between the retention of the antibodies and BMI. Our data may indicate that people with high BMI are a better indication for boost vaccination. On the other hand, our result showed that hypertension was the significant independent factor for the reduction rate and the presence of hypertension led to the slower decline. However, this is difficult to discuss from an immunological perspective.

Adverse reactions to vaccination by HD patients have also been reported, and according to Polewska et al. mild-to-moderate injection site pain is the most common reaction after the first and second vaccinations in HD patients, while the most common systemic reactions are fatigue, myalgia, and arthralgia. Previous report showed that many local and systemic adverse reactions were observed less frequently in HD patients, even though they were age- and sex- matched [19]. However, our data showed that the first vaccination did not significantly differ in the occurrence of adverse reactions between Control and HD groups, while after the second vaccination systemic symptoms of fever and nausea were significantly higher in HD group. Zitte et al. reported that the lower incidence of adverse reactions in HD patients than in Control group can be attributed in part to the younger age of Control group than that of HD group [20]. Therefore, it is possible that the age matching in this study allowed for a more accurate assessment of the incidence of adverse effects between HD patients and control, i.e., avoiding the bias of higher incidence of adverse effects in Control due to their younger age. Systemic adverse reactions are more common after the second vaccination in the HD group because HD patients are less likely to acquire antibodies after the first vaccination, and antibody titers tend to increase after the second vaccination, which may have increased the number of patients with fever after the second vaccination. Our study demonstrated that patients who had fever after the second vaccination had significantly higher anti-S1 antibody titers than those who did not have fever in both Control group and HD group. Therefore, fever may be observed in those who had a large increase in antibody titers after the second vaccination. In previous report, healthy participants with fever after the second vaccination have been shown to have significantly higher spike IgG titers than those without fever [21]. It is thought that, also in HD patients, fever reflects strong innate immune response and may be an indicator of elevated antibody titers.

As limitation, in our study, Control group consisted of those who were nursing home residents and outpatients at the hospital, which might not be a typical control group. In addition, the study was conducted in subjects who had not been infected with SARS-CoV-2, and whose antibody titers were confirmed as negative before the first dose of vaccine, but cases of asymptomatic infection within 3 months after the second vaccination could not be excluded. Furthermore, anti-S1 antibody titers were measured at the same time points in the HD group and Control group. However, Kitamura et al. reported more than half of the elderly HD patients showed an increase in their anti-spike IgG titers from 2 to 3 weeks after the second vaccination, although 84% of the healthy control group showed a decrease during the same time period [22]. Therefore, HD patients, especially in elderly, might have the slower rate of antibody titer compared to Control, and peak antibody titers may not be accurately evaluated at 2 weeks after the second dose of the vaccination.

## Conclusion

HD patients have significantly lower anti-S1 antibody titers after full vaccination than age- and sex-matched non-dialysis individuals. Dialysis time was identified as a factor affecting SARS-CoV-2 IgG titers in HD group, with longer dialysis time resulting in higher maximum SARS-CoV-2 IgG titers. Systemic reactions after vaccination in dialysis patients suggest an effective immune response.

## Data Availability

The datasets generated and/or analyzed during the current study are available from the corresponding author upon reasonable request.

## Abbreviations

COVID-19: Coronavirus disease 2019
SARS-CoV-2: Severe acute respiratory syndrome coronavirus type 2
HD: Hemodialysis
JSDT: Japanese Society for Dialysis Therapy
BMI: Body mass index
TP: Total protein
Alb: Albumin
AST: Aspartate aminotransferase
ALT: Alanine aminotransferase
ALP: Alkaline phosphatase
γ-GTP: γ-Glutamyl transpeptidase
BUN: Blood urea nitrogen
Cre: Creatinine
UA: Uric acid
IgG: Immunoglobulin
Fe: Iron
TIBC: Total iron binding capacity
CRP: C-reactive protein
HbA1c: Hemoglobin A1c
WBC: White blood cell
Hb: Hemoglobin
DM: Diabetes mellitus
COPD: Chronic obstructive pulmonary disease
